# USP36 promotes tumorigenesis and tamoxifen resistance in breast cancer by deubiquitinating and stabilizing ERα

**DOI:** 10.1186/s13046-024-03160-2

**Published:** 2024-08-31

**Authors:** Ting Zhuang, Shuqing Zhang, Dongyi Liu, Zhongbo Li, Xin Li, Jiaoyan Li, Penghe Yang, Chenmiao Zhang, Jiayao Cui, Mingxi Fu, Fangyu Shen, Lei Yuan, Zhao Zhang, Peng Su, Jian Zhu, Huijie Yang

**Affiliations:** 1 Xinxiang Key Laboratory of Tumor Migration and Invasion Precision Medicine, School of Medical Technology, Xinxiang Medical University, Xinxiang, 453003 Henan Province P.R. China; 2 Henan Key Laboratory of Immunology and Targeted Therapy, School of Medical Technology, Xinxiang Medical University, Xinxiang, 453003 Henan Province P.R. China; 3 Department of Anaesthesiology, The Second Hospital, Cheeloo College of Medicine, Shandong University, Jinan, 250033 Shandong Province P.R. China; 4 Department of General Surgery, The Second Hospital, Cheeloo College of Medicine, Shandong University, Jinan, 250033 Shandong Province P.R. China; 5 School of International Education, Xinxiang Medical University, Xinxiang, 453003 Henan Province P.R. China; 6 The Fifth Clinical Medical College of Henan University of Chinese Medicine (Zhengzhou People’s Hospital), Zhengzhou, 450003 Henan Province P.R. China; 7 Department of Pathology, Qilu Hospital, Cheeloo College of Medicine, Shandong University, Jinan, 250033 China; 8 Department of General Surgery, Shengjing Hospital of China Medical University, Shenyang, Liaoning Province 110000 P.R. China

**Keywords:** USP36, ERα, Breast cancer, Ubiquitin, Stability, Tamoxifen resistance

## Abstract

**Background:**

Breast cancer is the most prevalent cancer in women globally. Over-activated estrogen receptor (ER) α signaling is considered the main factor in luminal breast cancers, which can be effectively managed with selective estrogen receptor modulators (SERMs) like tamoxifen. However, approximately 30–40% of ER + breast cancer cases are recurrent after tamoxifen therapy. This implies that the treatment of breast cancer is still hindered by resistance to tamoxifen. Recent studies have suggested that post-translational modifications of ERα play a significant role in endocrine resistance. The stability of both ERα protein and its transcriptome is regulated by a balance between E3 ubiquitin ligases and deubiquitinases. According to the current knowledge, approximately 100 deubiquitinases are encoded in the human genome, but it remains unclear which deubiquitinases play a critical role in estrogen signaling and endocrine resistance. Thus, decoding the key deubiquitinases that significantly impact estrogen signaling, including the control of ERα expression and stability, is critical for the improvement of breast cancer therapeutics.

**Methods:**

We used several ER positive breast cancer cell lines, DUB siRNA library screening, xenograft models, endocrine-resistant (ERα-Y537S) model and performed immunoblotting, real time PCR, RNA sequencing, immunofluorescence, and luciferase activity assay to investigate the function of USP36 in breast cancer progression and tamoxifen resistance.

**Results:**

In this study, we identify Ubiquitin-specific peptidase 36 (USP36) as a key deubiquitinase involved in ERα signaling and the advancement of breast cancer by deubiquitinases siRNA library screening. In vitro and in vivo studies showed that USP36, but not its catalytically inactive mutant (C131A), could promote breast cancer progression through ERα signaling. Conversely, silencing USP36 inhibited tumorigenesis. In models resistant to endocrine therapy, silencing USP36 destabilized the resistant form of ERα (Y537S) and restored sensitivity to tamoxifen. Molecular studies indicated that USP36 inhibited K48-linked polyubiquitination of ERα and enhanced the ERα transcriptome. It is interesting to note that our results suggest USP36 as a novel biomarker for treatment of breast cancer.

**Conclusion:**

Our study revealed the possibility that inhibiting USP36 combined with tamoxifen could provide a potential therapy for breast cancer.

**Supplementary Information:**

The online version contains supplementary material available at 10.1186/s13046-024-03160-2.

## Background

There are approximately 1 million new cases of breast cancer worldwide each year, which is the most prevalent malignancy among women [1]. There are four types of breast cancer according to molecular pathology: luminal A, luminal B, HER2 and triple negative (TNBC) [2]. Both Luminal A and B type breast cancers are characterized by being Estrogen receptor α (ERα) positive. ERα, a nuclear receptor, plays a significant role in breast cancer development and advancement by controlling cell transformation, proliferation, and metastasis [3]. Breast cancer and estrogen signaling have been linked for more than 80 years. In 1985, ESR1 was cloned, which is the major driver of the oncogenic process in luminal breast cancers. The ERα protein consists of 595 amino acids, which include trans-activation domain 1 (AF1), Ligand Binding Domain (LBD), and DNA binding domain (DBD) [4]. Tamoxifen, a selective ER modulator, effectively targets the ER signaling pathway for both preventing and treating breast cancer in patients with ERα-positive breast cancer [5]. Tamoxifen, which shares similar structure with estradiol, competes with estrogen for DNA binding and blocks ERα target gene expression [6]. The emergence of endocrine resistance is an urgent clinical issue that will inevitably occur in approximately 30–40% of breast cancer patients [7]. Numerous regulation models, including protein post-translational modifications (PTMs), have been proposed in several studies to elucidate the mechanisms underlying endocrine resistance [8].

Ubiquitination is a widespread protein post-translational modification that plays a crucial role in regulating various cellular functions [9]. The ubiquitination process is reversible and can be catalyzed by a series of deubiquitylating enzymes termed deubiquitinases (DUB) [10]. According to the current knowledge, approximately 100 deubiquitinases are encoded in the human genome, but it remains unclear which deubiquitinases play a critical role in estrogen signaling and endocrine resistance [11]. Therefore, to detect deubiquitinating enzymes potentially involved in ER signaling, we conducted a genome-wide siRNA screen targeting DUB enzymes. The purpose of this study is to identify key deubiquitinases that significantly impact breast cancer progression and that may have important implications for treatment options against tamoxifen resistance.

In our study, we found that USP36 interacts with ERα, leading to the suppression of ERα polyubiquitination and degradation in breast cancer cells, which indicated that USP36 linked to breast cancer proliferation and invasion via estrogen signaling.

## Materials and methods

### Cell lines and cell culture

The MCF-7, T47D, and HEK293T cell lines, acquired from the American Type Culture Collection (ATCC), were cultured in Dulbecco’s modified Eagle’s medium (DMEM, D6429, Sigma-Aldrich) supplemented with 10% fetal bovine serum (FBS, 10,270–106, Gibco), 4.5 g/L glucose, and 4 mM L-glutamine. The cells were incubated in charcoal-stripped FBS (Gibco, 12,676–029) and treated with phenol red-free DMEM (Gibco, 11,330–057) for experiments involving E2. All cell line authentication was performed through short tandem repeat (STR) analysis, and the STR data for the MCF-7, T47D, and HEK293T cell lines matched the data provided by ATCC.

### Plasmids and siRNA

The Myc-USP36, Flag-USP36 and USP36 deletion mutants (residues 1–420; 421–800 and 801–1121) plasmid were a kind gift from Wenhao Zhang [12]. In the previous study [13, 14], HA-ERα, Flag-ERα, ERα deletion mutants (residues 1–180, 1–300, 180–595, and 300–595), as well as HA-Ub, HA-K48, and HA-K48R plasmids were utilized. According to the instructions of the manufacturer, Lipofectamine 2000 (1,662,298, Invitrogen) was used for plasmids transfection. When the cells were 40–60% confluent, Lipofectamine RNAi MAX (3778–075, Invitrogen) was used for siRNA transfection. The sequence of siRNA synthesized from GenePharma (China) used are listed in Supplementary Table 1.

### Lentivirus-mediated knockdown of USP36 expression

The lentiviral shUSP36 vectors were generated via ligation of hybridized oligos into the PLKO.1 lentiviral vector (linearized with BsmBI) using T4 DNA ligase (NEB). The sequence of the shRNA targeting USP36 used are listed in Supplementary Table 1. The lentiviral vectors were co-transfected with the psPAX2 and pMD2.G into HEK293T cells using Lipofectamine 2000 (Invitrogen). Medium containing viral particles was collected 48 h after transfection and passed through a 0.45 μM filter. MCF-7 cells were transduced with viral supernatant supplemented with 8 μg/mL polybrene (Sigma–Aldrich). Stably transfected cells were selected with 6 μg/ml puromycin (Solarbio).

### DUB siRNA library screening

Human Deubiquitinating Enzyme (ON-TARGET plus) was obtained from Dharmacon siRNA library (GU-104705). Transfection of ERα-positive breast cancer cells MCF-7 with various siDUBs for 48 h resulted in the isolation of RNA, which was then reverse-transcribed into cDNA. The expression levels of the ERα classical downstream gene, TFF1(PS2), were analyzed to investigate the potential modulation of the ERα signaling pathway. The real time PCR results of siControl were normalized to 1. The primer sequences for real time PCR are listed in Supplementary Table 2.

### RNA extraction and real time PCR analysis

The manufacturer’s instructions were followed to extract cellular RNA using RNeasy Plus Mini Kit (Tiangen, DP451). The extracted RNA was reverse transcribed using HiScript II Q RT SuperMix (Vazyme, R223-01) following the manufacturer’s instructions. Quantitative real-time polymerase chain reaction was then conducted on a 7500 Fast Real-Time PCR System (Applied Biosystems, Singapore) using SYBR real-time PCR Master Mix (Vazyme, Q511-02). 36B4 was used as the internal reference gene. An analysis of the 2^−∆∆Ct^ method was used to determine the relative expression levels. Sangon Biotech composed the primers used in this study, which are listed in Supplementary Table 2.

### Western blotting

To conduct Western blotting, cells were harvested and lysed with Western and IP Lysis Buffer (P0013J, Beyotime) supplemented with protease and phosphatase inhibitors (Thermo). Using Bradford protein assays, the concentration of proteins was determined. Subsequently, 20–30 µg of protein was separated using SDS–polyacrylamide gel electrophoresis (PAGE), followed by the transfer of proteins to PVDF membranes (Millipore). Primary antibodies utilized. Following three washes with PBST, secondary antibodies (Beyotime, A0216, 1:6000 or Beyotime, A0208, 1:6000) were applied. Western blotting analysis was performed using the specified antibodies. The antibody used are listed in Supplementary Table 3. Signals were visualized using Western blotting substrate from ECL.

### Coimmunoprecipitation (Co-IP) assay

To conduct coimmunoprecipitation assays, 500 µg of protein lysate was precleared with 20 µl of Protein A + G Agarose (Beyotime, P2028) and rabbit IgG (Beyotime, A7016, 1:50) for 2 h at 4 °C. Immunoprecipitation was subsequently carried out for 4 h at 4 °C with the specified antibody. Negative controls included either rabbit IgG (Beyotime, A7016, 1:50) or mouse IgG (Beyotime, A7028, 1:50). Western blotting analysis was performed using the specified antibodies. The antibody used are listed in Supplementary Table 3.

### Luciferase activity assay

The estrogen signaling luciferase activity was measured with the Dual-Luciferase Reporter kit from Promega in Germany. Cells were transfected with the ERE luciferase reporter along with Renilla. Based on the manufacturer’s instructions, the activity of luciferase was measured 24 h post-treatment.

### CCK8 assay

An assay for cell viability was performed using the Cell Counting Kit (CCK-8), 4 × 10^3^ cells were plated into 96-well plates. The proliferation levels were assessed at designated time interval. By using a multifunctional enzyme-linked analyzer (BioTek, USA), the optical density (OD) value at 450 nm was quantified.

### Colony formation assay

MCF-7 and T47D cells were transfected in 24-well plates using a combination of 20 nM USP36 siRNA or 20 nM siControl. After two weeks, the assays were performed as described previously [15].

### Transwell assay

The migration assay was conducted in uncoated Transwell chambers with 8 µm pore size (Corning, USA). Firstly, the upper chamber was inoculated with 5 × 10^4^ cells suspended in 200 µl of serum-free medium, while the lower chamber was inoculated with 500 µl of medium containing 20% FBS. Following a 24-h incubation period, 4% paraformaldehyde and Hematoxylin were used to fix and stain the cells that crossed the lower surface of the membrane. Subsequently, cells from three randomly selected fields were counted in triplicate for the experiment.

### Wound healing assay

Transfecting MCF-7 and T47D cells with siUSP36 or siControl in a 6-well plate until confluence was performed was used for the wound healing assay. Subsequently, a linear scratch was created using a 200 µl yellow sterile tip gun along a straight edge. The scratch closure was monitored and photographed microscopically at designated time intervals post-scratch initiation. The separation between the two boundaries was measured and contrasted with the original distance at the specified time.

### Apoptosis analysis

In the apoptosis assay, MCF-7 and T47D cells were stained with Annexin V and propidium iodide (PI) using Vazyme’s Annexin V-FITC Apoptosis Detection Kit (#A211-02). Fluorescence intensity was quantified using a FACScan (Millipore), and data were analyzed with the Flowjo7.6 software using forward scatter (FSC).

#### Protein stability assay

The half-life of endogenous or ectopically expressing ERα was determined using a Cycloheximide (CHX) chase assay. Cells were transfected with 20 nM siControl/siUSP36 or 1 μg Flag/Flag-USP36 WT/Flag-USP36 Mutants, cultured in 12-well plates. The cells were treated with 100 µM of cycloheximide (C7698, Sigma) for specified durations after 48 h. Next, ER degradation was detected using immunoblotting and western blotting of equal amounts of boiled lysates.

### Poly-ubiquitination detection assay

K48-linked poly-ubiquitination as an example, K48-linked poly-ubiquitination of ERα was detected in cell extracts by co-transfecting cells with Myc-USP36 or Myc tagged with K48-Ub plasmids and Flag-ERα plasmids. Following a 24-h transfection period, cells were exposed to a concentration of 10 μM MG132 for a duration of 6 h. Protein extraction followed, followed by preclearance with protein A for 2 h. Anti-ERα or anti- Flag antibodies were then incubated overnight with the extract, followed by an hour of incubation with protein A/G beads at 4 °C. Ultimately, the western blotting was performed using anti-HA antibodies to detect the level of K48 polyubiquitinated ER.

### Immunofluorescence assay

Coverslips were placed in 12-well cell culture plates with MCF-7 cells. Immediately after incubating for 24 h, the MCF-7 cells on the coverslips were fixed with 4% paraformaldehyde for 15 min, permeabilized with 0.25% Triton X-100 for 15 min and blocked with 5% BSA for 1 h. Subsequently, the coverslips were incubated with primary antibodies specific to USP36 and ERα at 4 °C for an overnight duration. After rinsing with PBS, the coverslips were exposed to a secondary antibody labeled with a fluorophore (Invitrogen), and DAPI (Beyotime) was employed for staining the cell nuclei. Negative controls were prepared by incubating samples solely with secondary antibodies, omitting primary antibodies. A Nikon A + laser scanning confocal microscope was used to capture images, and ImageJ software was used for image processing and assembly.

### Analyses of public clinical data

The tumor RNA-seq data for breast cancer from USP36 can be obtained via the Genomic Data Commons (GDC) data portal website. Prism 9.0 software was utilized for data analysis and calculations. Additionally, USP36 expression was analyzed in ER-positive, ERα-negative breast cancer tissues, and normal tissues derived from the TCGA database. The association between USP36 expression and clinical prognosis was investigated through analysis utilizing the KMPLOT database (https://kmplot.com).

### Data analysis of RNA sequencing

The RNA-sequencing data are provided in GEO database (GSE262678). Pathway analysis of differentially expressed genes (DEGs) with a statistical significance threshold of P value < 0.05 and fold change > 1.5 was conducted using hallmark gene sets and KEGG pathways in Metascape (https://metascape.org). A visualization of the findings was created in the form of a heatmap using the freely available data analysis and visualization platform located at http://www.bioinformatics.com.cn. Subsequently, a volcano plot showing the differentially expressed genes (DEGs) that satisfy the set criteria (P < 0.05 and fold change > 2) was generated using the OmicStudio tools found at https://www.omicstudio.cn/tool. The gene set enrichment analysis (GSEA) used the HALLMARKS_ESTROGEN_RESPONSE_LATE gene sets from the Molecular Signatures Database v7.4. The Gene Set Enrichment Analysis (GSEA) was conducted utilizing the GSEA 4.1.0 software with the standard default settings.

### Xenograft mouse models

Female M-NSG mice, aged four weeks, were implanted with slow-release 17 beta-estradiol pellets (0.72 mg/90-day, obtained from Innovative Research of America) upon acquisition from Shanghai Model Organisms Center, Inc. A total of 6 mice were included in each group. Approximately 4 × 10^6^ MCF-7 cells together with Matrigel solution were injected into the mammary fat pad of each mouse. Subsequently, seven-day intervals were then used to measure tumor sizes. The tumor volume was determined as width squared multiplied by length, then divided by 2. The Ethics Committee of Xinxiang Medical University approved all procedures involving animal subjects. Throughout the study, the mice were housed in a controlled environment, maintained at a specific pathogen-free (SPF) level with regulated temperature and lighting conditions (12 h light/12 h dark cycle), and free access to food and water.

### Statistics

The statistical analyses employed in this study included the student’s t-test and Pearson correlation coefficient using publicly available data. Data were presented as the mean ± standard error of the mean (SD), with statistical significance defined as *P* < 0.05 (*), *P* < 0.01 (**), and *P* < 0.001 (***).

## Results

### Identification of USP36 as a novel mediator of ER signaling in ER positive breast *cancer*

**Fig. 1:**
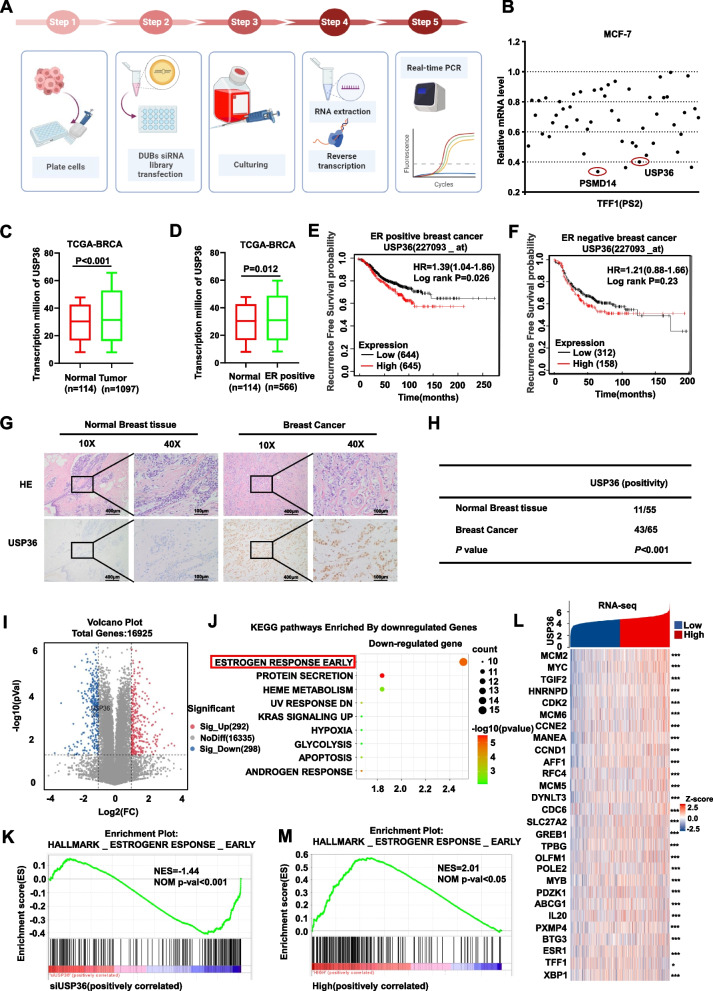
Identification of USP36 as a novel mediator of ER signaling in ER positive breast cancer. **A** The flowchart shows the siRNA screening procedure for identifying novel deubiquitinases involved in modulating ER signaling. Each of the 76 human DUB genes was knocked down in MCF-7 cells with 20 μM pooled siRNAs. After 48 h, the quantitative gene expression analysis was detected by real-time PCR. **B** The relative expression level of TFF1(PS2) in MCF-7 cells transfected with DUBs in the screening library. The real-time PCR results of siControl is normalized to 1. **C** The expression of USP36 in breast cancer tissues (*n* = 1097) and normal tissues (*n* = 114) from TCGA database (https://www.genome.gov/). **D** The expression of USP36 in ER positive breast cancer tissues (*n* = 566) and normal tissues (*n* = 114) from TCGA database (https://www.genome.gov/). **E**–**F** Kaplan − Meier analysis showing relapse-free survival depending on USP36 expression levels in ER positive and ER negative breast cancer from public meta-analysis data (https://kmplot.com). **G-H** Immunohistochemistry (IHC) detecting USP36 expression in 65 breast cancer samples and 55 normal breast tissue (**G**). Statistical analysis of USP36 expression in G (**H**). **I** Volcano map of RNA-seq data from MCF-7 cell lines treated with siControl or siUSP36. |log2Fold change|> 1 and *P* value < 0.05 are set as screening criteria. **J** KEGG analysis of downregulated genes in RNA-seq data from MCF-7 cell lines treated with siControl or siUSP36 with threshold criteria of P < 0.05 and fold change > 1.5. **K** Gene set enrichment analysis (GSEA) shows enrichment of estrogen response genes in RNA-seq data from MCF-7 cell lines treated with siControl or siUSP36. **L** Heatmap of relationship of USP36 and differentially ER pathway related genes in RNA-seq data with threshold criteria of *P* < 0.05 and fold change > 1.5. **M** Gene set enrichment analysis (GSEA) shows enrichment of estrogen response genes in breast cancer tissue from TCGA data with threshold criteria of P < 0.05 and fold change > 1.5. All P values were calculated by unpaired two-tailed Student’s t tests. *P < 0.05; **P < 0.01; ***P < 0.001. (**C-L**)

We employed a siRNA screen targeting genome-wide DUB enzymes to detect deubiquitinating enzymes which can regulate ER signaling activity, we did the siRNA screening using the DUBs siRNA library (Dharmacon Company, Cat: G104705) in MCF-7 cells (Fig. 1A). TFF1 (PS2) is a well-established target gene of ERα [16]. To assess ER signaling activity, we used PS2 as an indicator. Analysis of the data revealed that depleting USP36 notably reduced PS2 mRNA levels in MCF-7 cells (Fig. 1B). We also uncovered that the knockdown of several other DUBs such as PSMD14 and USP1 dramatically reduced PS2 mRNA expression which have reported in our previous studies [13, 17]. We further examined the expression of USP36 in human breast cancer and found that both the mRNA and protein level of USP36 were increased in breast malignancies based on data from the TCGA database (Fig. 1C and Fig. S1A). Furthermore, both the mRNA and protein levels of USP36 was also elevated in breast cancer samples that were positive for ERα (Fig. 1D and Fig. S1B). Meanwhile, we used a breast healthy control cell line MCF-10A as a control to compare differences of USP36 in mRNA and protein levels by real-time PCR and immunoblot analysis. The result indicted that USP36 upregulated in ER-positive cell lines both at the transcriptional level and protein level (Fig. S1C-D). In addition, this conclusion was reinforced and consistent with the results of Sun XX et al. [18]. Additionally, we investigated the association between USP36 and breast cancer survival using data from the TCGA database (https://kmplot.com/analysis/). Our analysis revealed that high USP36 levels were linked to shorter survival rates in ERα positive breast cancer patients (Fig. 1E). While further analysis revealed that USP36 was not associated with survival in ERα negative breast cancer patients, suggesting that the survival impact of USP36 may depend on ER status (Fig. 1F). We also assessed the protein level of USP36 in breast cancer patient samples using immunohistochemistry (IHC), interestingly, Breast cancers also showed significant increases in USP36 expression (11/55 vs 43/65; *P* < 0.001; Fig. 1G-H) which was consistent with the result from TCGA database (Fig. 1C). In addition, we further depleted USP36 in MCF-7 cells for RNA sequencing analysis (GSE262678). The volcano plot showed that there are 590 changed (Fig. 1I). Hallmark gene set and KEGG pathway analysis in Metascape (https://metascape.org) revealed that USP36 depletion impacts various aspects of cancer biological processes. Interestingly, in terms of downregulated signaling pathways, estrogen signaling ranked first (Fig. 1J). Furthermore, GSEA (Gene Set Enrichment Analysis) in indicated that the HALLMARK_ESTROGEN RESPONSE gene set were globally decreased in USP36 depletion condition (NES = -1.44; *P *< 0.001; Fig. 1K). In addition, the heat map analysis of RNA sequencing showed that depleting USP36 effectively reduced the mRNA expression of classical ERα target genes, including TFF1, GREB1, PDZK1 and IL20 (Fig. 1L). In addition, GSEA indicated that the HALLMARK_ESTROGEN RESPONSE gene set signature was significantly enrichment in the presence of USP36 high expression based on the expression in breast cancer tissue from TCGA database (NES = 2.01; *P* < 0.05; Fig. 1M). Based on these results, we suggest that USP36 may serve as a potential enhancer of ER signaling in breast cancer.

### USP36 depletion inhibits ERα positive breast *cancer* progression in vivo and in vitro

**Fig. 2:**
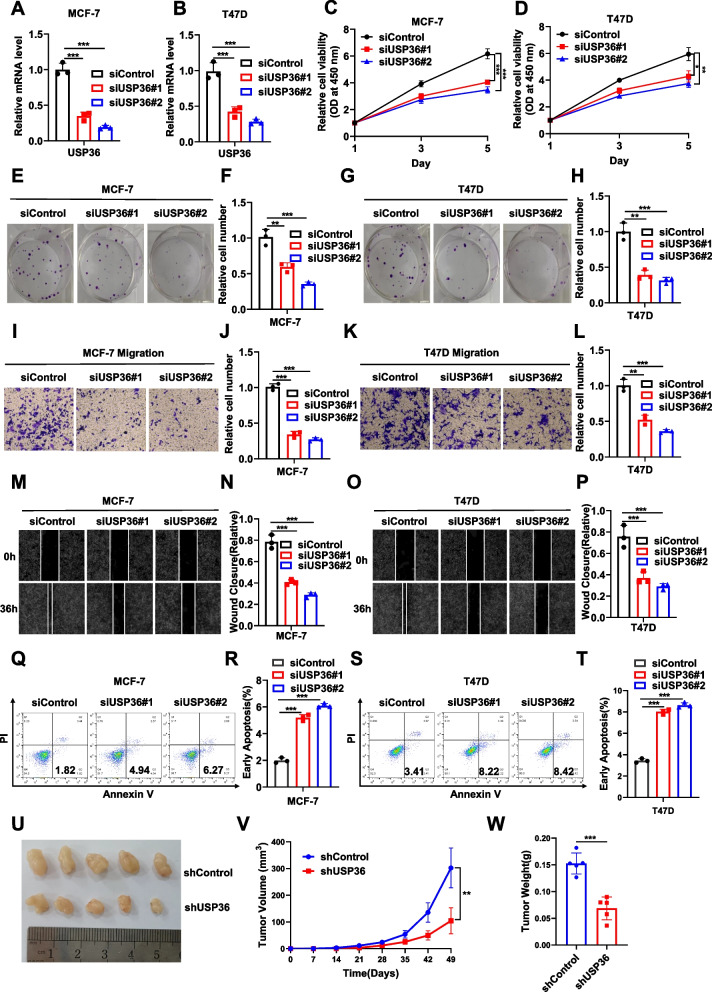
USP36 depletion inhibits ERα positive breast cancer progression in vivo and in vitro*. ***A-B** Real-time PCR was performed to determine USP36 mRNA levels in MCF-7 and T47D cells following USP36 siRNA treatment 48 h. **C-D** MCF-7 and T47D cells transfected with siControl or siUSP36 for 48 h were tested for viability using the CCK-8 assay at the indicated time points. **E**–**H** Colony formation (left panel) of MCF-7 and T47D cells transfected with scrambled siRNA or two independent USP36 siRNAs for 48 h. F and H show the quantitative analysis of the colony formation assay results. **I**-**L** MCF-7 and T47D cells were tested for their migration ability using Transwell assays. J and L show the quantitative analysis of the Transwell assays results. **M**-**P** MCF-7 and T47D cells were tested for their migration ability using wound healing assays. **N** and **P** show the quantitative analysis. **Q-T** The percentage of apoptotic cells was determined by FACS analysis after MCF-7 and T47D cells were treated with USP36 siRNA for 48 h. PI and Annexin V staining were performed on the cells. **U** A representative image of a tumor derived from a nude mouse injected with stably transfected shControl or shUSP36 MCF-7 cells is shown. **V-W** The tumor volume (**V**) and weight (**W**) in nude mice subcutaneously inoculated with stably transfected shControl or shUSP36 MCF-7 cells.Three independent experiments were conducted to obtain the results shown in Panels **A-W**. All the data are presented as the means ± SDs. *P < 0.05; **P < 0.01; ***P < 0.001 for comparisons (Student’s t test)

We further investigated the influence of USP36 on the ER-positive breast cancer phenotype by reducing USP36 levels with siUSP36 or shUSP36 method. shControl or shUSP36 MCF-7 cells were stably transduced by lentivirus. USP36 depletion was confirmed using real-time PCR (Fig. 2A-B) and Immunoblot analysis (Fig. S2A), and significantly impaired the proliferation of ER-positive breast cancer cells, as indicated by the CCK8 assay. (Fig. 2C-2D and Fig. S2B). Furthermore, the colony formation assay showed that reducing USP36 decreased colony formation capacity (Fig. 2E-H and Fig. S2C-D). We further examined the expression level of USP36 protein every five days, and the results showed that USP36 protein expression could also downregulated in the day 15 following siRNA performed (Fig.S3A). Meanwhile the Transwell assay revealed that USP36 depletion reduced the migration capacity of MCF-7 and T47D cells (Fig. 2I-L and Fig. S2E-F). In addition, this conclusion was reinforced by the results of the wound-healing assay (Fig. 2M-P and Fig. S2G-H). Additionally, we analyzed the impact of USP36 on apoptosis. Propidium iodide (PI)/Annexin V staining demonstrated that depleting USP36 resulted in higher proportions of apoptotic cells (Fig. 2Q-T and Fig. S2I-J). In addition, a subcutaneous xenograft tumorigenesis model was created by randomly dividing nude mice into two groups and treating them with shControl or shUSP36 MCF-7 cells stably transduced by lentivirus through subcutaneous injection. The xenograft mouse model verified that silencing USP36 in MCF-7 cells suppressed tumor growth in vivo (Fig. 2U-W). Overall, USP36 significantly contributes to the advancement of ERα positive breast cancer.

### USP36 instead of USP36 C131A overexpression promotes ERα positive breast *cancer* progression in vivo and in vitro

**Fig. 3:**
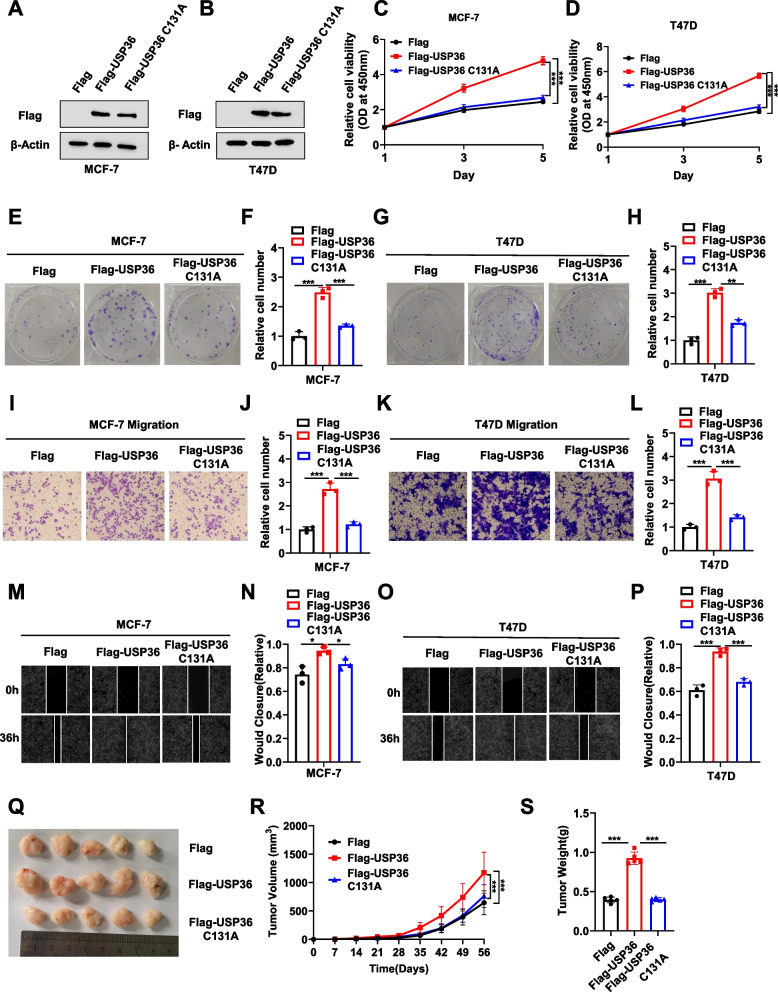
USP36 instead of USP36 C131A overexpression promotes ERα positive breast cancer progression in vivo and in vitro*. ***A-B** Immunoblot analysis showing the expression level of USP36 in MCF-7 and T47D cells transfected with Flag or Flag-USP36 WT or Flag-USP36 C131A plasmid. β-Actin was used as the internal control. **C**-**D** MCF-7 and T47D cells transfected with Flag or Flag-USP36 WT or Flag-USP36 C131A plasmid for 48 h were tested for viability using the CCK-8 assay at the indicated time points. **E**–**H** Colony formation (left panel) of MCF-7 and T47D cells transfected with indicated plasmid for 48 h. F and H show the quantitative analysis of the colony formation assay results. **I-L** MCF-7 and T47D cells transfected with indicated plasmid for 48 h, were tested for their migration ability using Transwell assays. J and L show the quantitative analysis of the Transwell assays results. **M-P** MCF-7 and T47D cells transfected with indicated plasmid for 48 h, were tested for their migration ability using wound healing assays. N and P show the quantitative analysis. **Q** A representative image of a tumor derived from a nude mouse injected with stably MCF-7 cells as indicated is shown. **R-S** The tumor volume (**R**) and weight (**S**) in nude mice subcutaneously inoculated with stably transfected MCF-7 cells as indicated. Three independent experiments were conducted to get the results shown in Panels **C-S**. All the data are presented as the means ± SDs. *P < 0.05; **P < 0.01; ***P < 0.001 for comparisons (Student’s t test)

In order to further examined the impact of USP36 on the ER positive breast cancer phenotype, by overexpressing of USP36 wild-type (WT) plasmid, and USP36 catalytically inactive mutant (C131A) [19], We found that USP36 WT and USP36 C131A was successfully achieved, as validated by western blotting (Fig. 3A**-**B). The CCK8 assay indicated USP36 instead of its catalytically inactive mutant (C131A) overexpression significantly promoted ER positive breast cancer cell proliferation (Fig. 3C**-**D). In addition, the colony formation assay showed that USP36 overexpression increased the colony formation capacity but not its C131A (Fig. 3E**-**H). Transwell assay also indicated that USP36 instead of USP36 C131A overexpression increased the migration capacity of MCF-7 and T47D cells (Fig. 3I**-**L). Moreover, in the wound-healing assay, wound closure speed sharply increased in both MCF-7 and T47D cells with USP36 instead of USP36 C131A overexpression (Fig. 3M**-**P). Moreover, the xenograft mouse model confirmed that overexpression of USP36 WT, compared to USP36 C131A, can increase tumor growth in vivo. (Fig. 3Q-S).

### USP36 instead of USP36 C131A is required for ERα signaling in breast *cancer*

**Fig. 4:**
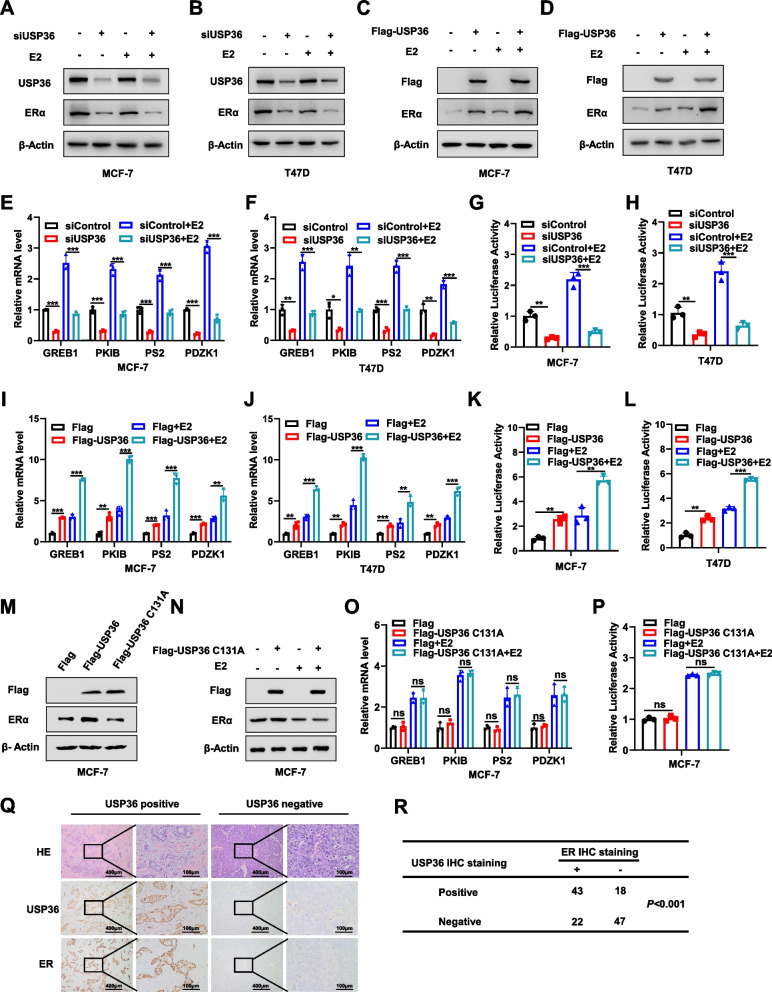
USP36 instead of USP36 C131A is required for ERα signaling in breast cancer. **A-B** USP36 and ERα protein levels were determined by western blotting. MCF-7 and T47D cells in charcoal-stripped FBS and phenol red-free DMEM were transiently transfected with 20 nM siControl or 20 nM siUSP36 and then treated with 10 nM estradiol or vehicle for 6 h. Cell lysates were immunoblotted with the indicated antibodies. β-Actin was used as internal control. **C-D** USP36 and ERα protein levels were determined by western blotting. MCF-7 and T47D cells in charcoal-stripped FBS and phenol red-free DMEM were transiently transfected with Flag or Flag-USP36 for 48 h, and then treated with 10 nM estradiol or vehicle for 6 h. Cell lysates were immunoblotted with the indicated antibodies. β-Actin was used as internal control. **E**–**F**;** I-J**;** O** GREB1, PKIB, PS2 and PDZK1 mRNA levels were determined by real-time PCR after treatment with indicated method in MCF-7 and T47D cells. **G-H**;** K**-**L**;** P** ERE-luciferase activity was detected by luciferase assays in MCF-7 and T47D cells. **M**–**N** USP36 and ERα protein levels were determined by western blotting. MCF-7 cells were transiently transfected with Flag or Flag-USP36 WT or Flag-USP36 C131A plasmid for 48 h. β-Actin was used as the internal control. **Q-R** Immunohistochemical (IHC) staining to evaluate USP36 and ERα expression in HCC tissues. In Panels **E-R**, the results are representative of three independent experiments. All the data are presented as the means ± SDs. *P < 0.05; **P < 0.01; ***P < 0.001 (Student’s t test)

As ERα is crucial in estrogen signaling, we investigated the impact of USP36 on ERα protein. Depleting USP36 reduced ERα protein levels in both vehicle and estradiol-treated conditions (Fig. 4A**-**B). Additionally, the real time PCR data showed that silencing USP36 inhibited the expression of ERα target genes, including GREB1, PKIB, PS2 and PDZK1 in not only vehicle-treated but also estradiol-treated circumstance (Fig. 4E**-**F and Fig. S2K). In addition, we conducted an estrogen response element (ERE) luciferase assay in MCF-7 and T47D cells to investigate the impact of USP36 knockdown on ERα transcriptional activity. Our findings revealed a significant reduction in ERE luciferase activity following USP36 depletion (Fig. 4G**-**H). Conversely, overexpression of USP36 in MCF-7 and T47D cells enhanced expression of ERα protein, ERα signaling target genes and signaling activity under both vehicle and estradiol treatment circumstance (Fig. 4C**-**D, I**-**L). Furthermore, we also indicated that USP36 catalytically inactive mutant (C131A) have no effect on ERα signaling (Fig. 4M**-**P). Additional immunohistochemistry analysis of 65 breast cancer samples demonstrated a positive correlation between USP36 and ERα (Fig. 4Q**-**R).

### USP36 promotes breast *cancer* progression via ERα

**Fig. 5:**
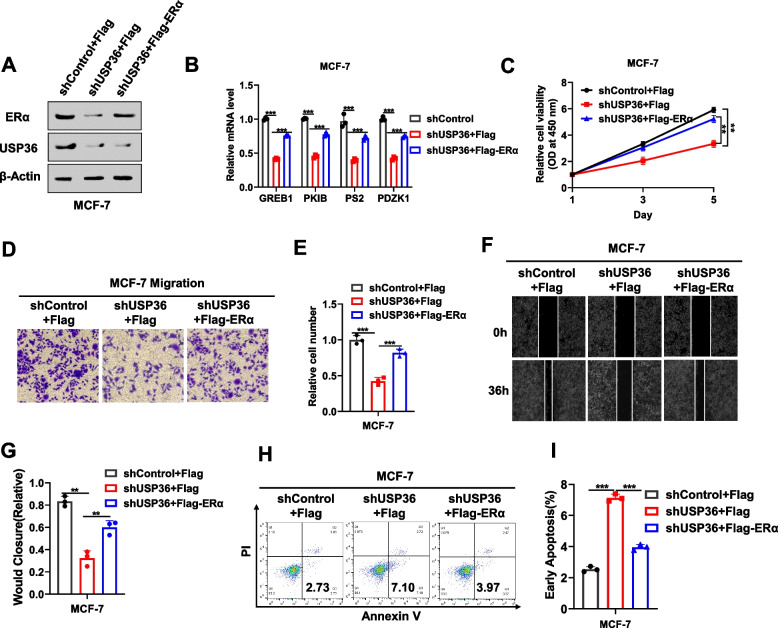
USP36 promotes breast cancer progression via ERα. **A** Immunoblot analysis showing the expression level of ERα and USP36 in MCF-7 cells stably transduced with shUSP36, transfected with Flag or Flag-ERα plasmid. β-Actin was used as the internal control. **B** GREB1, PKIB, PS2 and PDZK1 mRNA levels were determined by real time PCR after treatment with Flag or Flag-ERα plasmid for 48 h in MCF-7 cells stably transduced with shUSP36. **C** MCF-7 cells stably transduced with shUSP36 were transfected with Flag or Flag-ERα plasmid for 48 h. The CCK-8 assays were used to detect the cell viability at the indicated time points. **D-E** MCF-7 cells stably transduced with shUSP36 were transfected with Flag or Flag-ERα plasmid for 48 h. Transwell assays were used to detect migration ability. E shows the quantitative analysis of the Transwell assays results. **F-G** MCF-7 cells stably transduced with shUSP36 were transfected with Flag or Flag-ERα plasmid for 48 h. Wound healing assays were used to detect migration ability. G shows the quantitative analysis. **H-I** MCF-7 cells stably transduced with shUSP36 were transfected with Flag or Flag-ERα plasmid for 48 h. The percentage of apoptotic cells was determined by FACS analysis. PI and Annexin V staining were performed on the cells. I shows the quantitative analysis. Three independent experiments were conducted to get the results shown in Panels **A-I**. All the data are presented as the means ± SDs. **P < 0.01; ***P < 0.001 for comparisons (Student’s t test)

To inquire whether ERα is contained in USP36-mediated proliferation, migration and apoptosis properties of breast cancer, we carried out several rescue experiments. The effectiveness of USP36 silencing by stably transduced with indicated shUSP36 via lentivirus and ERα overexpression was substantiated through western blot analysis (Fig. 5A). Additionally, the real time PCR data showed that shUSP36 also significantly inhibited the expression of ERα target genes, including GREB1, PKIB, PS2, and PDZK1, which could be mostly rescued by further ERα overexpression (Fig. 5B). Furthermore, the CCK8 assay indicated that USP36 depletion resulted in the inhibition of breast cancer cell proliferation, a phenotype that was partially rescued upon further ERα overexpression (Fig. 5C). Similarly, the results obtained from the transwell and wound healing assays suggested that the migratory capacity of breast cancer cells was notably hampered after USP36 deprivation. Nevertheless, this inhibitory effect on cell migration was partially reversed by subsequent overexpression of ERα (Fig. 5D-G). The apoptosis assay indicated USP36 silencing increased the proportion of apoptotic cells, which effect could be partially rescued by further ERα overexpression (Fig. 5H-I). Overall, these results indicated that ERα partially accounts for the anti-tumor effect of USP36 depletion.

### USP36 associates with ERα and modulates ERα stability in breast *cancer* cells

**Fig. 6:**
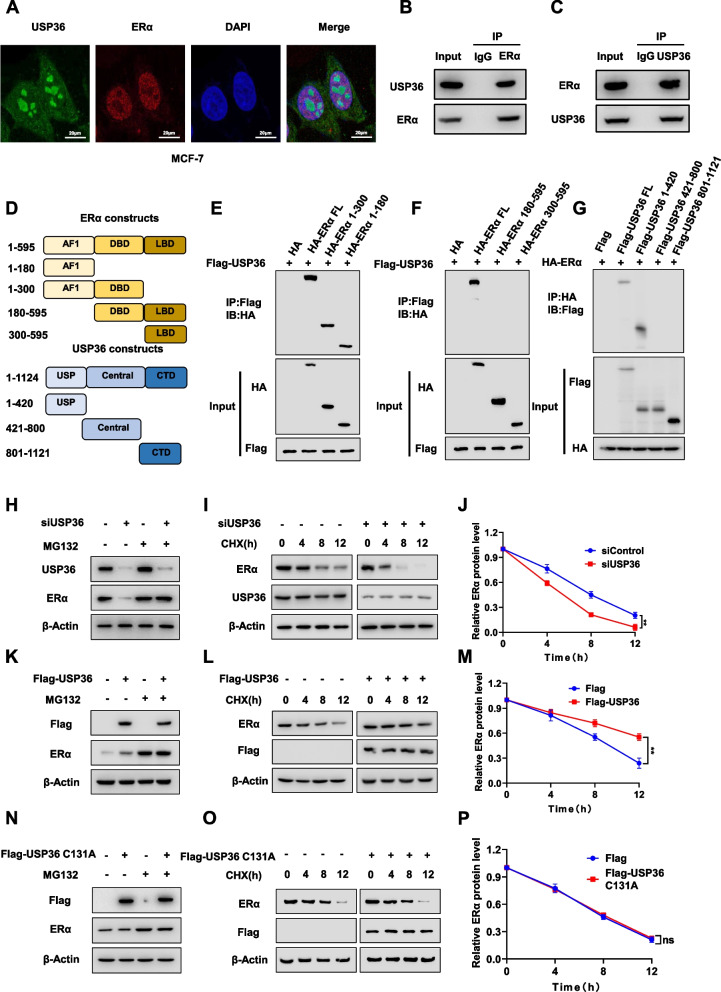
USP36 associates with ERα and modulates ERα stability in breast cancer cell. **A** Immunofluorescence staining assay showing the localization patterns of USP36 and ERα in MCF-7 cells. Intracellular localization of USP36 (green) and ERα (red) is shown. Nucleus (blue) were stained with DAPI. Scale bar, 20 µM. **B-C** Immunoprecipitation assay showing the endogenous interaction between USP36 and ERα. For examining the endogenous interaction between USP36 and ERα, lysates of MCF-7 cells were precipitated with anti-ERα or anti-USP36 antibodies, and the precipitates were examined by immunoblotting with 2% input sample. **D** Schematic of the ERα protein, along with the ERα deletion mutants (residues 1–180, 1–300, 180–595 and 300–595) used in the Co-IP assays. Schematic of the USP36 protein, along with the USP36 deletion mutants (residues 1–420; 421–800 and 801–1121) used in the Co-IP assays. **E**–**F** Immunoprecipitation assay showing AF1 domain is required for ERα to interact with USP36. HEK293T cells were co-transfected with 2 µg USP36 plasmid and full-length HA-ERα or mutant ERα (1–180, 1–300, 180–595 and 300–595). After 24 h, the cells were treated with 10 μM MG132 for 6 h. Then, the cells were harvested with NP-40 lysis buffer. Co-IP was performed using an anti-Flag antibody, and the possible interacting ERα domains were detected with anti-HA antibody. **G** Immunoprecipitation assay showing USP36 interacts with ERα through its USP domain (1–420). Co-IP was performed using an anti-HA antibody, and the possible interacting USP36 domains were detected with anti-Flag antibody. **H**;** K**;** N** USP36 and ERα protein levels were determined by western blotting. Cells by treat with 10 μM proteasome inhibitor MG132 for 6 h. **I-J**;** L**-**M**;** O**-**P** USP36 and ERα protein levels were determined by western blotting. MCF-7 cells were treated with 100 μM cycloheximide (CHX) for the indicated times. The expression of ERα protein was estimated by ImageJ software and is represented graphically in the right panel (**J**, **M**, **P**). In Panels **A-C**, **E**-**I**, **K-L**, **N**–**O**, the results are representative of three independent experiments. All the data are presented as the means ± SDs. *P < 0.05; **P < 0.01 (Student’s t test)

We further examined the localization of USP36 and ERα in breast cancer cells using an immuno-staining assay. The results indicated that ERα predominantly localized in the nucleus, whereas USP36 was found both in the cytosol and nucleus (Fig. 6A). The immuno-precipitation assay in MCF-7 cells revealed that USP36 interacts with ERα (Fig. 6B-C). Several studies have reported the ERα protein consists of three functional domains: AF1, DBD, and LBD [20]. While the USP36 protein comprises the USP (deubiquitinase) domain, central domain, and CTD (C-terminal domain) (Fig. 6D). Deletion constructs were created to study the interaction between USP36 and ERα. The findings showed that the USP domain of USP36 is required for its interaction with ERα, while the AF1 domain of ERα is required for its interaction with USP36 (Fig. 6E-G). The study further investigated the biological impact of USP36 on ERα protein. Depletion of USP36 resulted in a decrease in ERα protein levels in MCF-7 cells, which was reversed by the proteasome inhibitor MG132 (Fig. 6H). Furthermore, the protein stability assay with the protein synthesis inhibitor cycloheximide showed that depleting USP36 in MCF-7 cells reduces the half-life of ERα protein (Fig. 6I-J). Consistent with this finding, overexpression of USP36, as opposed to USP36 C131A, in MCF-7 cells increased the level of ERα protein, and this function was minimized by MG132 treatment (Fig. 6K, N) and lengthened ERα protein half-life (Fig. 6L-M, O-P). These data indicates that the impact of USP36 on ERα stability relies on deubiquitinase activity.

### USP36 regulates ERα protein stability by suppressing K48-linked polyubiquitination of ERα protein

**Fig. 7:**
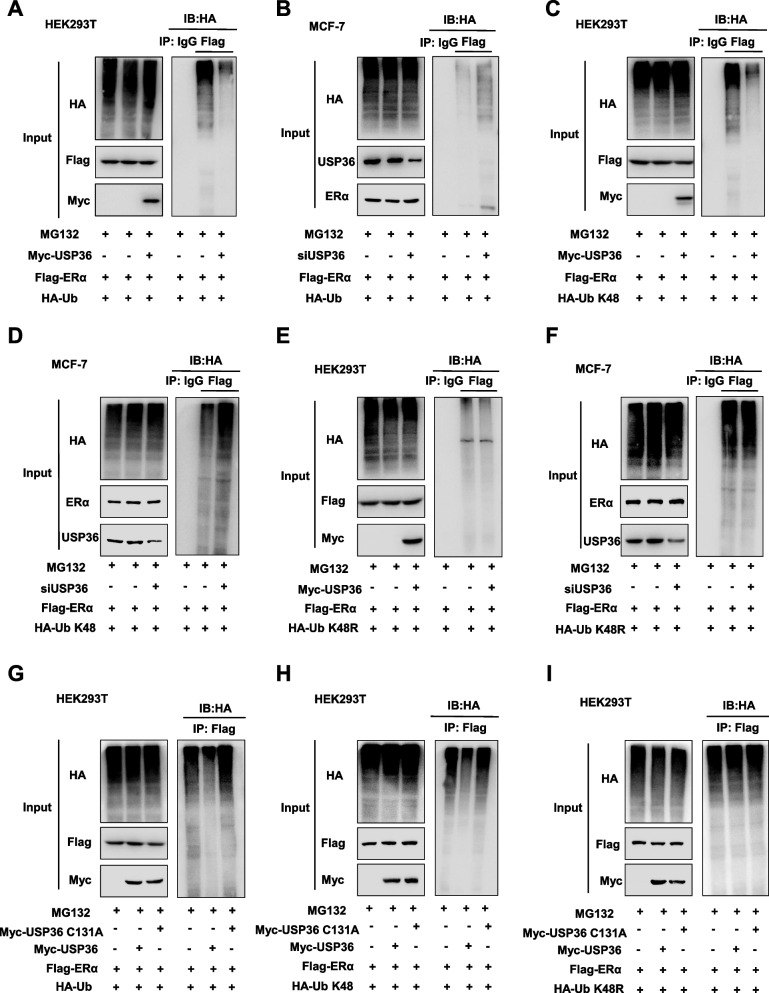
USP36 regulates ERα protein stability by inhibiting K48-linked polyubiquitination of ERα. **A** Polyubiquitinated ERα was detected via western blotting. HEK293T cells were co-transfected with 1 µg Flag-ERα plasmid, 0.5 µg HA-Ub plasmid and 0.5 µg Myc-tag or Myc-USP36 plasmids, plasmids in HEK293T cells upon MG132 treatment and then immunoblotted with the indicated antibodies. **B** Polyubiquitinated ERα was detected via western blotting. MCF-7 cells were transfected with 1 µg Flag-ERα plasmid, 0.5 µg HA-Ub plasmid and 20 μM USP36 siRNA upon MG132 treatment for 6 h and then immunoblotted with the indicated. **C-D** K48-specific polyubiquitinated ERα was detected via western blotting in HEK293T and MCF-7 cells with indicated antibodies. **E**–**F** K48R-specific polyubiquitinated ERα was detected via western blotting in HEK293T and MCF-7 cells with indicated antibodies. **G** Total polyubiquitinated ERα was detected via western blotting in HEK293T cells with indicated antibodies. **H** K48-specific polyubiquitinated ERα was detected via western blotting in HEK293T cells with indicated antibodies. **I** K48R-specific polyubiquitinated ERα was detected via western blotting in HEK293T cells with indicated antibodies. All the results are representative of three independent experiments

USP36, a deubiquitinating enzyme within the USP family [21], was investigated for its potential role in regulating the polyubiquitination of the ERα protein. Experimental findings using a polyubiquitination assay in HEK293T cells showed that overexpressing USP36 led to a reduction in the total polyubiquitination level of ERα (Fig. 7A). In addition, depleting USP36 in MCF-7 cells increased the total polyubiquitination level of ERα through endogenous proteins (Fig. 7B). Next, we investigated the specific subtypes of ubiquitin chains that are implicated in modifying ERα and are under the regulation of USP36. K48-linked ubiquitination stands out as the most prevalent degradation mechanism among the various ubiquitination mechanisms [22]. Interestingly, our research demonstrated that USP36 decreased K48-linked ubiquitination specifically (Fig. 7C). Furthermore, we validated these findings through ubiquitin-based immunoprecipitation assays in MCF-7 cells. (Fig. 7D). Moreover, the mutant of ubiquitin (K48R) was found to lessen the function of USP36 on ERα poly-ubiquitination, as illustrated in Fig. 7E**-**F. This finding affirms that USP36 targets the K48-linked ubiquitination of ERα specifically. However, USP36 catalytically inactive mutant (C131A) overexpression could not decreased the total and K48 polyubiquitination level of ERα (Fig. 7G-I), which suggest that the effect of USP36 on K48-linked polyubiquitination of ERα relies on the activity of deubiquitinase.

### Inhibiting USP36 could restore tamoxifen sensitivity in a model of endocrine-resistant breast *cancer*

**Fig. 8:**
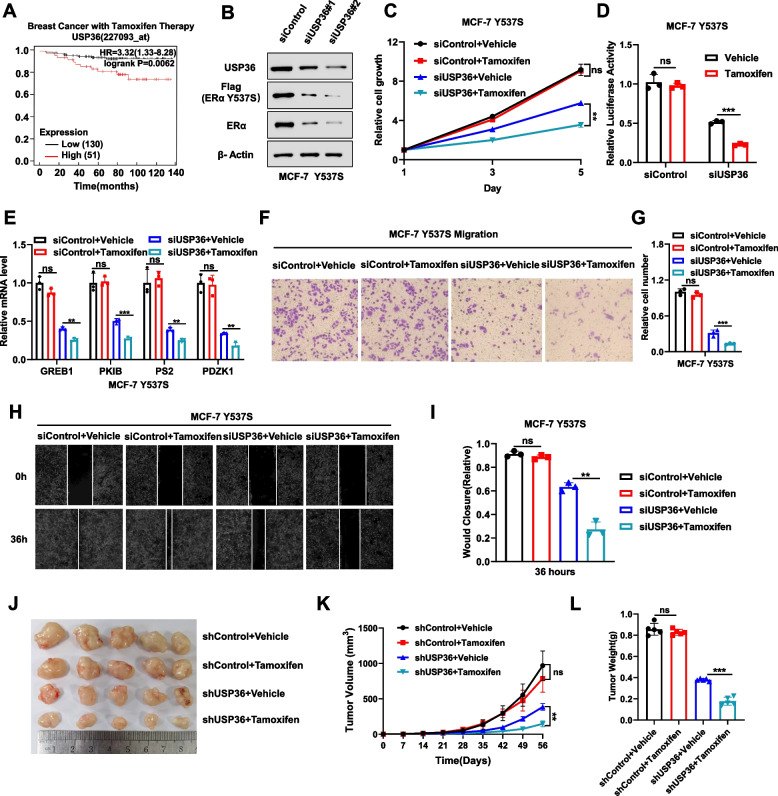
USP36 inhibition could restore tamoxifen sensitivity in endocrine resistant breast cancer model. **A** The Kaplan–Meier analysis conducted on data sourced from the meta-analysis available at (https://kmplot.com) revealed a significant correlation between elevated levels of USP36 expression and decreased survival rates among breast cancer patients undergoing tamoxifen treatment. **B** USP36 depletion reduces the protein level of ERα and ERα Y537S in MCF-7 Y537S cells. Immunoblotting of cell lysates will be performed using specific antibodies with β-actin as the loading control. **C** Depleting USP36 restores tamoxifen’s inhibitory effect on MCF-7 Y537S cells. Cells will be transfected with siControl or siUSP36 for 24 h, treated with 1 μM tamoxifen for 12 h, and cell metabolic activity will be measured using the CCK-8 assay at specified time points. **D** Luciferase assay demonstrates that USP36 depletion can restore the inhibitory effect of tamoxifen on luciferase activity in MCF-7 Y537S cells. **E** Depleting USP36 restores tamoxifen’s inhibitory effect on ERα target genes in MCF-7 Y537S cells. Cells will be transfected with siControl or siUSP36 for 24 h, then treated with 1 μM tamoxifen for 12 h. RNA will be extracted for gene expression analysis, with each group analyzed in triplicate. **F-G** Transwell assay demonstrates that USP36 depletion reduces the migratory ability of MCF-7 Y537S cells. **H-I** Wound healing assay demonstrates that USP36 depletion reduces the migratory ability of MCF-7 Y537S cells. **J-L** In a xenograft model, deletion of USP36 restored the inhibitory effect of tamoxifen on MCF-7 Y537S. Tumor growth was monitored in vivo in different groups of mice by measuring tumor growth (**J**), tumor volume (**K**) and weight (**L**). Three independent experiments were conducted to get the results shown in Panels C-L. All the data are presented as the means ± SDs. *P < 0.05; **P < 0.01; ***P < 0.001 for comparisons (Student’s t test)

We established a stable MCF-7 cell line expressing the mutant ERα (Y537S) to create an endocrine-resistant model. Common mutations found in tamoxifen-resistant breast cancer patients involve Y537C/S/N in the ligand-binding domain of ERα [23]. Using this model, we assessed how USP36 influences the breast cancer phenotype and ERα signaling in an endocrine-resistant context. We further investigated the role of USP36 in breast cancer survival with tamoxifen therapy using data from the TCGA database (https://kmplot.com/analysis/). Interestingly, the survival data indicated a negative correlation between high USP36 expression and survival outcomes in breast cancer patients undergoing tamoxifen therapy (Fig. 8A). The immuno-blotting data revealed that depleting USP36 reduced levels of both wild type and mutant forms of ERα (Fig. 8B). The CCK8 assay showed that depleting USP36 restored tamoxifen’s inhibitory effect in breast cancer cells (Fig. 8C). We also indicated that the depletion of USP36 could restore the inhibitory effect of tamoxifen on ERα signaling activity, as evidenced by the luciferase assay (Fig. 8D). The real time PCR data showed that depleting USP36 restored tamoxifen’s inhibitory effect on ERα target genes, including GREB1, PKIB, PS2, and PDZK1 (Fig. 8E). In addition, the Transwell assay and wound-healing assay reinforced this conclusion (Fig. 8F-I). Furthermore, In the xenograft mice model, depleting USP36 hindered breast tumor growth and boosted tamoxifen’s inhibitory effect in the Y537S-expressing MCF-7 model (Fig. 8J**-**L). Therefore, targeting USP36 may overcome endocrine therapy resistance caused by mutant ERα.

## Discussion

**Fig. 9:**
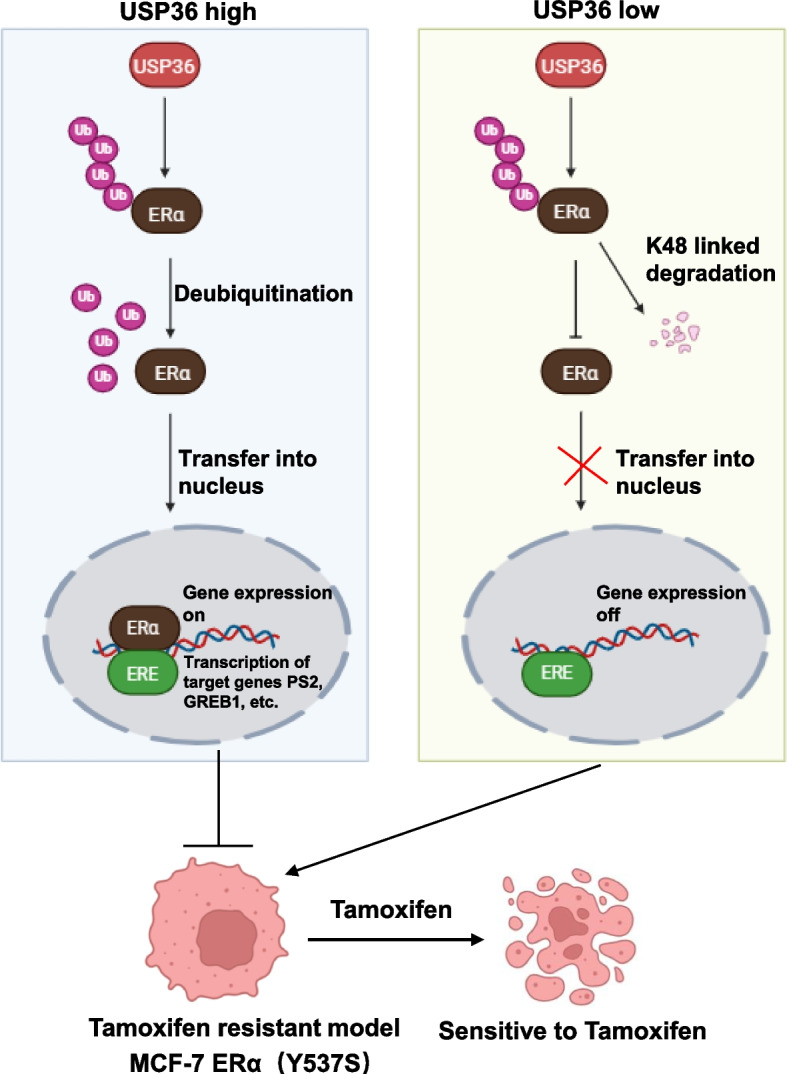
A hypothetical model of the mechanism of USP36 regulation of ERα signaling. The inhibition of USP36 decreases breast cancer progression via modulating ERα K48-linked deubiquitinating, which subsequently suppresses ERα signaling activity and tamoxifen resistance (draw by biorender)

This study reveals that the nucleolar deubiquitinating enzyme USP36 plays a key role in regulating ERα ubiquitination and tamoxifen resistance (Fig. 9). USP36 is correlated with the gene signature of ERα signaling, associates with poor survival in ER positive breast cancer and tamoxifen treatment. Mechanistically, we identified USP36 promoted breast cancer development by decreasing K48-linked ubiquitinating of ERα protein, thereby enhancing ERα signaling activity and tamoxifen resistance. Our findings underscore the central role of USP36 in regulating the ubiquitination of ERα, thereby increasing its stability. Further investigation confirmed that the interaction of USP36 and ERα promotes the deubiquitination of ERα. These results highlight the potential of USP36 as a therapeutic target in addressing tamoxifen resistance.

ERα has been identified as a promising prognostic biomarker and is crucial for guiding treatment of breast cancer [24]. Tamoxifen is a widely used treatment for patients with ER-positive breast cancer. Tamoxifen competitively blocks the binding of estrogen to ERα and cause ERα to take on a different shape that inhibits the binding of the coactivator. Unfortunately, tamoxifen resistance will eventually occur in 30–40% of breast cancer patients, posing an urgent clinical issue [25]. Multiple studies have proposed various regulatory models to elucidate tamoxifen resistance, including transformation into ERα negative status and the presence of constitutively active mutations in specific functional domains of ERα [26]. Nevertheless, in most cases of tamoxifen resistance, the ER remains expressed and activated, indicating the presence of other mechanisms that regulate ESR1 to confer tamoxifen resistance. Emerging evidence indicated that ESR1 expression was regulated by diverse aspects, including post-translational modifications (PTMs), histone modification, SUMOylation [27], somatic mutation, ESR1 fusion genes. Since breast cancer patients are faced with the challenge of endocrine resistance, so understanding the ERα signaling activity, including its expression and stability regulation are critical for the development of new anti-estrogen therapies and the defeating of tamoxifen resistance.

However, recent studies identified mutations at specific sites in the gene that encodes ERα in a large subset of patients with breast cancers that have spread. These mutations make ERα resistant to antiestrogen drugs. The most prevalent ERα point mutations were Y537S [28]. We established a stable MCF-7 cell line expressing the mutant ERα (Y537S) to create an endocrine-resistant model. We firstly carried out the experiments to check if USP36 depletion could affect ERα level (both WT and Y537S). The data showed that USP36 depletion could reduce the protein level of ERα in both WT and Y537S form. The logic is if ERα level is reduced, the basal ER signaling activity is partially inhibited, which means less tamoxifen concentration is need to achieve satisfiable inhibition effect for ER signaling and cellular phenotype in ER + cells.

Ubiquitination is a widespread protein post-translational modification (PTM) [29]. Recent studies have revealed increasing evidence of the impact of E3 ubiquitin ligases on ERα signaling effect and tamoxifen sensitivity [30]. Our previous research demonstrated that SMURF1, TRIM56 and RNF181 can interact with ERα and enhance breast cancer growth [14, 15, 31]. The process of deubiquitination by DUBs, the reversed course of ubiquitin–proteasome system-mediated proteolysis [32], has been shown to greatly impact various cellular processes, including immune reactions, drug resistance and so on. According to the current knowledge, approximately 100 deubiquitinases are encoded in the human genome [33]. Previous studies demonstrated that USP7 [34] and USP37 [35] could remove theK48-ubiquitin chain from ERα, leading to inhibited proteasome-mediated ERα degradation. However, further research is still required to explore the exact deubiquitinases play a critical role in estrogen signaling and endocrine resistance. Therefore, to detect deubiquitinating enzymes potentially involved in ER signaling, we conducted a genome-wide siRNA screen targeting DUB enzymes. Additionally, we discovered that USP36 positively associated with genes downstream of the ER signaling and correlates with shorten survival in ER positive breast cancer.

USP36, a member of the ubiquitin-specific protease (USP) family, is a deubiquitinating enzyme. Recent research suggested USP36 plays a pivotal role in various cellular biological processes by deubiquitinating proteins [36]. This process results in the reduction of proteasomal degradation of proteins involved in tumorigenesis, immune responses, cell cycle progression, and autophagy [37]. Our previous research showed that USP36 plays a crucial role in regulating Hippo signaling activity by inhibiting YAP/TAZ K48-linked polyubiquitination [12, 38]. Furthermore, in this study we indicated that USP36 facilitate breast cancer development via adjusting K48-linked deubiquitinating of ERα protein, and then enhancing ERα signaling activity. What’s more, we also revealed elevated levels of USP36 are associated with reduced survival rates in breast cancer patients undergoing tamoxifen therapy. Furthermore, in the model of endocrine resistant breast cancer cells, inhibiting USP36 could recover tamoxifen sensitivity in vivo and in vitro.

However, there were limitations to this study, even though our study mainly concentrated on the role that USP36 plays in modulating ubiquitination of ERα and tamoxifen resistance. Research on inhibitors targeting ubiquitin-specific proteases has yielded a range of outcomes, including USP1, USP5, USP8, USP7, USP15, USP25/28, and USP30 [39]. It is not currently possible to find inhibitors that specifically target USP36. Hopefully, future research endeavors will lead to the development of drugs that selectively degrade USP36 or USP36 inhibitors to alleviate the potential for tamoxifen resistance. Despite the lack of such drugs currently available, we are optimistic that future research will address this gap in the field.

In conclusion, our results demonstrate that USP36 modifies the ubiquitination of estrogen receptors and tamoxifen resistance, which may provide a new therapeutic target for the treatment of breast cancer. A novel modulator of estrogen signaling modulation of USP36 activity or gene expression level may be an attractive treatment option for breast cancer. Additionally, Further investigations are necessary to understand how DUBs function in ERα and in tamoxifen resistance, as well as to assess their potential as therapeutic candidates in various models of tamoxifen resistance.

### Supplementary Information

Supplementary Material 1.

Supplementary Material 2: Supplementary Figure 1: A The protein expression of USP36 in breast cancer tissues (*n=*125) and normal tissues (*n=*18) from TCGA database (https://www.genome.gov/). B: The protein expression of USP36 in ER positive breast cancer tissues (*n=*64) and normal tissues (*n=*18) from TCGA database (https://www.genome.gov/). C: Immunoblot analysis showing the expression level of USP36 in MCF-7, T47D and MCF-10A cells. β-Actin was used as the internal control. D: Real-time PCR results of USP36 mRNA expression in MCF-7, T47D and MCF-10A cells exposed as indicated. Three independent experiments were conducted to obtain the results shown in Panels C-D. All the data are presented as the means ± SDs. ***P < 0.001 for comparisons (Student’s t test). Supplementary Figure 2. A: Immunoblot analysis showing the expression level of USP36 in MCF-7 cells stably transduced with indicated shRNAs by lentivirus. Immunoblotting analyses were performed using the indicated antibodies. β-Actin was used as the internal control. B: The CCK-8 assays were used to detect the cell viability of MCF-7 cells stably transduced with indicated shRNAs by lentivirus at the indicated time points. C-D: Colony formation (left panel) of MCF-7 cells stably transduced with indicated shRNAs by lentivirus. E shows the quantitative analysis of the colony formation assay results. E-F: Transwell assays were used to detect migration ability of MCF-7 cells stably transduced with indicated shRNAs by lentivirus. G shows the quantitative analysis of the Transwell assays results. G-H: Wound healing assays were used to detect migration ability of MCF-7 cells stably transduced with indicated shRNAs by lentivirus. I shows the quantitative analysis. I-J: The percentage of apoptotic cells was determined by FACS analysis of MCF-7 cells stably transduced with indicated shRNAs by lentivirus. PI and Annexin V staining were performed on the cells. K shows the quantitative analysis. K: GREB1, PKIB, PS2 and PDZK1 mRNA levels were determined by real-time PCR in MCF-7 cells stably transduced with indicated shRNAs by lentivirus. Three independent experiments were conducted to obtain the results shown in Panels A-K. All the data are presented as the means ± SDs. *P < 0.05; **P < 0.01;***P < 0.001 for comparisons (Student’s t test). Supplementary Figure 3A: USP36 protein levels were determined by Immunoblot analysis. MCF-7 cells were transiently transfected with 20 nM siControl or 20 nM siUSP36 for a total of 15 days. Protein was extracted every five days. Using Bradford protein assays, the concentration of proteins was determined. Then, cell lysates were immunoblotted with the indicated antibodies. β-Actin was used as an internal control. Three independent experiments were conducted to obtain the results.

## Data Availability

The publicly available data are provided in GEO database (GSE262678). The siRNA screening data, WB, teal time PCR original data, and cell line authentications were listed in the supplementary materials.

## Abbreviations

DUB: Deubiquitinase
USP36: Ubiquitin Specific Protease 36
UPS: Ubiquitin–Proteasome System
ERα: Estrogen Receptor α
AF1: Activation Functional Domain 1
LBD: Activation Functional Domain 2
DBD: DNA Binding Domain
GSEA: Gene set enrichment analysis
TNBC: Triple Negative Breast Cancer
HER2: Human epidermal growth factor receptor 2
PTM: Post-translational modification
