# Supplementary material for: USP36 promotes tumorigenesis and tamoxifen resistance in breast cancer by deubiquitinating and stabilizing ERα
Source: J Exp Clin Cancer Res. 2024 Aug 31;43:249. doi: 10.1186/s13046-024-03160-2 (PMC11365244; doi:10.1186/s13046-024-03160-2)
Supplement: Supplementary file 1 — Supplementary Material 1. [file 13046_2024_3160_MOESM1_ESM.docx]

**Supplemental information**

**Supplementary Table 1.** Sequences of siUSP36, shUSP36

| **Gene** | **Sequences** |
| --- | --- |
| siUSP36#1 | 5’-GCAAAUAUGUGUUGCUCAATT-3’  5’-UUGAGCAACACAUAUUUGCTT-3’ |
| siUSP36#2 | 5’-CCGGCAAGCUGCGAAUAUUTT-3’  5’-AAUAUUCGCAGCUUGCCGGTT-3’ |
| siControl | 5’-UUCUCCGAACGUGUCACGUTT-3’  5’-ACGUGACACGUUCGGAGAATT-3’ |
| shUSP36#1 | F: 5’-GATCGATCCGGCAAGCTGCGAATATTTCAAGACAATATTCGCAGCTTGCCGGATCTTTTT-3’  R: 5’-CGCGAAAAAGATCCGGCAAGCTGCGAATATTGTCTTGAAATATTCGCAGCTTGCCGGATC-3’ |
| shUSP36#2 | F: 5’-GATCGACTACCTTGGTCCATCAAATTTTCAAGACAAATTTGATGGACCAAGGTAGTTTTTT-3’  R: CGCGAAAAAACTACCTTGGTCCATCAAATTTGTCTTGAAAATTTGATGGACCAAGGTAGTC-3’ |

**Supplementary Table 2.** Primer sequences for qRT-PCR

| **Gene** | **Primer Sequences** |
| --- | --- |
| 36B4 | F: 5’-GCAGCATCTACAACCCTGAAG-3’  R: 5’-CACTGGCAACATTGCGGAC-3’ |
| GREB1 | F: 5’-CTGTACCACAGACGGGTTTTG-3’  R: 5’-TTCCGTGAAGTAACAGAAGCC-3’ |
| PKIB | F: 5’-GAGTCTGGGGTCGCCAATTTT-3’  R: 5’-TGAACTCTGGATGTCTGGTAAGG-3’ |
| USP36 | F: 5’-TTCGGCACAGCTGCTCTC-3’  R: 5’-CTCGGGGACAACAGCATCTT-3’ |
| PS2 | F: 5’-TGGGCTTCATGAGCTCCTTC-3’  R: 5’-TTCATAGTGAGAGATGGCCGG-3’ |
| PDZK1 | F:5’-TTCCTGCGAATTGAGAAGGAC-3’  R:5’-TCCACCCGTGTTTTCACTGC-3’ |

**Abbreviations:** F, forward; R, reverse.

**Supplementary Table 3.** Antibodies used in the present study.

| **Target** | **Source** | **No. of Catalogue** |
| --- | --- | --- |
| Anti-USP36(R) | Proteintech | 14783-1-AP |
| Anti-USP36(M) | Proteintech | 68165-1-Ig |
| Anti-ERα(R) | Cell Signaling Technology | 8644 |
| Anti-β-Actin(R) | Cell Signaling Technology | 3700 |
| Anti-HA(R) | Cell Signaling Technology | 3724 |
| Anti-UB(R) | Proteintech | 10201-2-AP |
| Anti-Flag(R) | Sigma–Aldrich | F9291 |
| Anti-Myc(R) | Abcam | Ab9106 |
| Rabbit IgG | Beyotime | A7016 |
| Mouse IgG | Beyotime | A7028 |
| Goat Anti-Rabbit IgG | Beyotime | A0208 |
| Goat Anti-Mouse IgG | Beyotime | A2016 |
| Protein A+G Agarose | Beyotime | P2028 |
