# Supplementary figures and images for: USP36 promotes tumorigenesis and tamoxifen resistance in breast cancer by deubiquitinating and stabilizing ERα
Source: J Exp Clin Cancer Res. 2024 Aug 31;43:249. doi: 10.1186/s13046-024-03160-2 (PMC11365244; doi:10.1186/s13046-024-03160-2)

Supplementary Figure 1

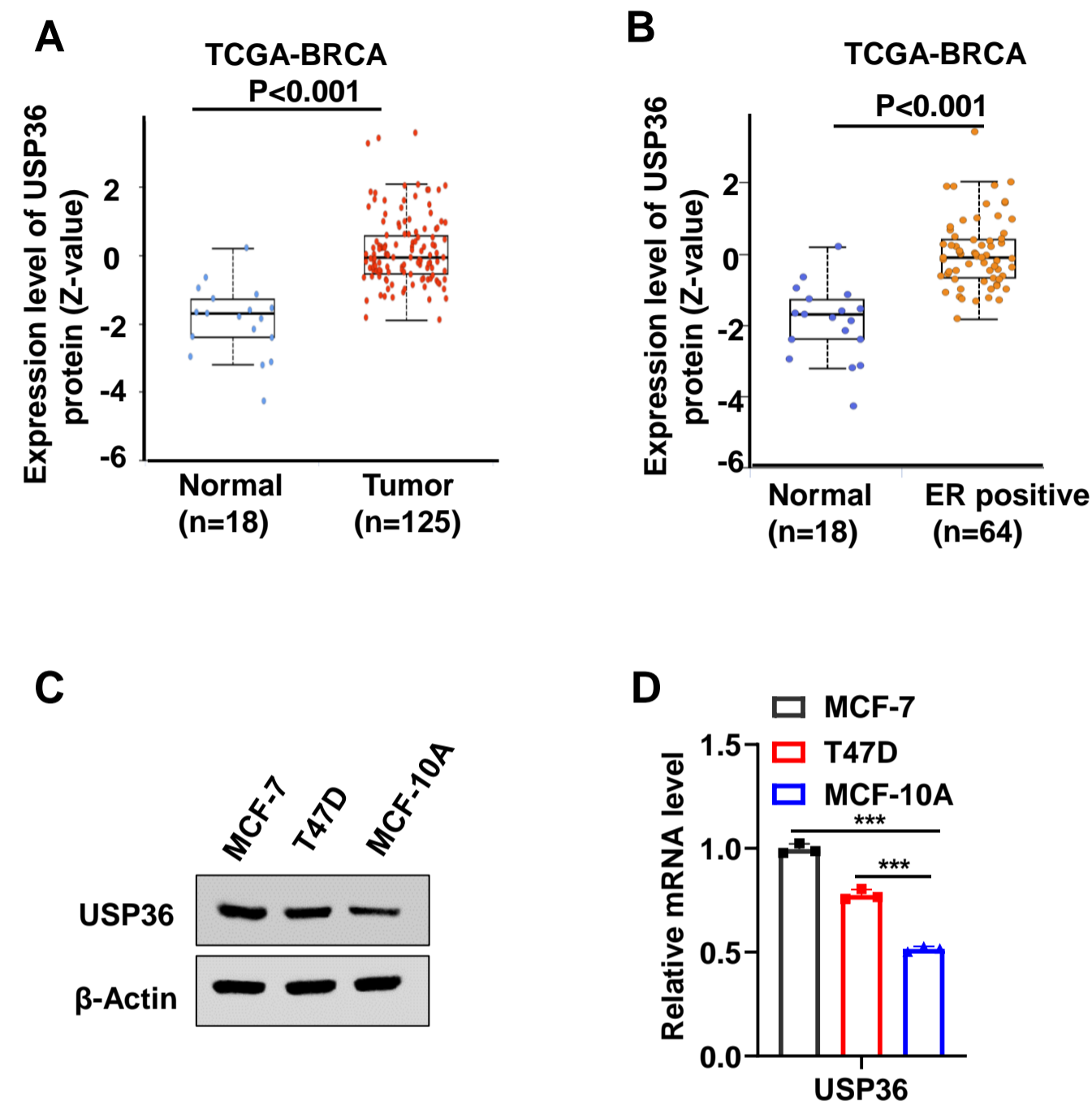

Supplementary Figure 2

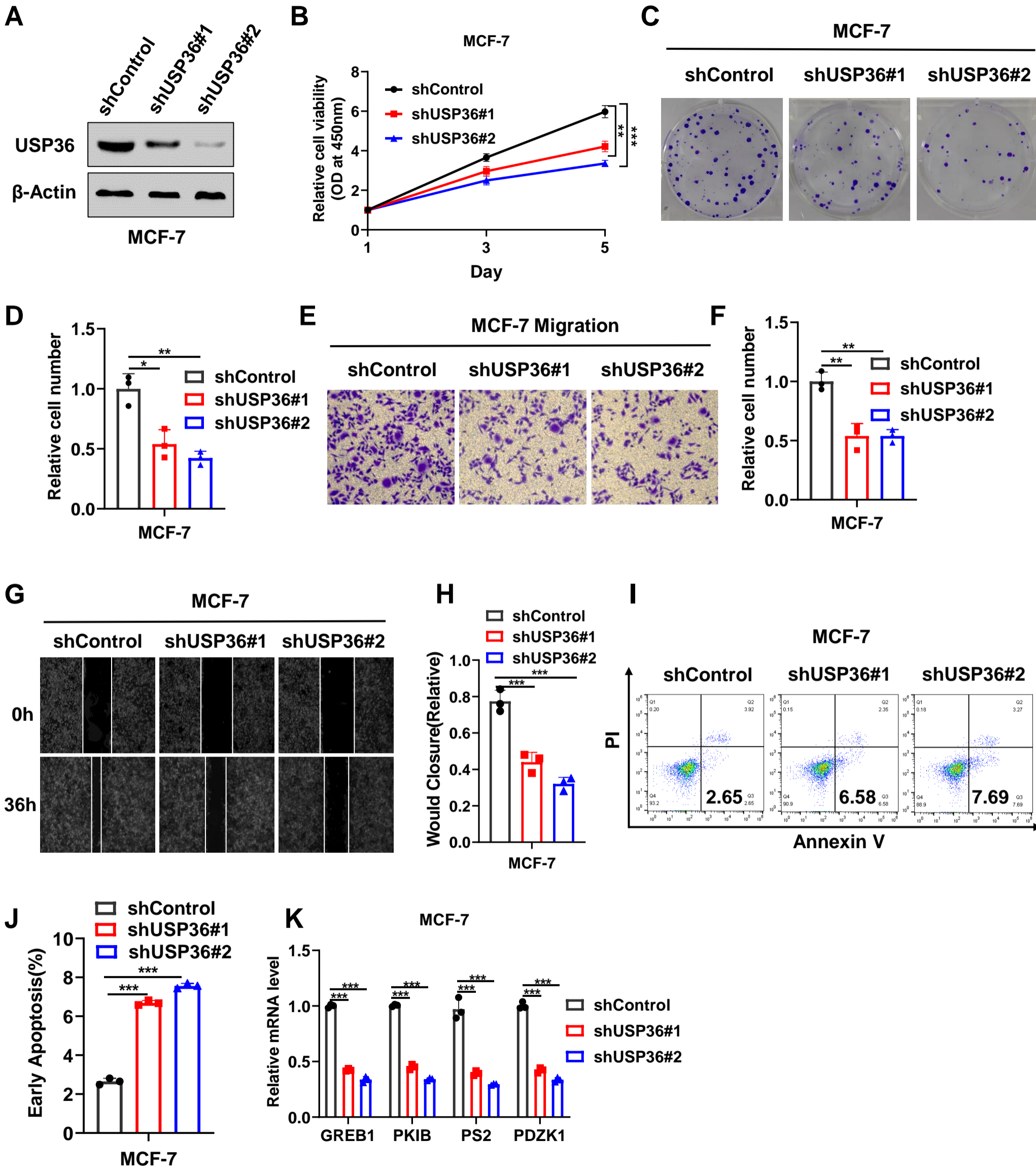

# Supplementary Figure 3

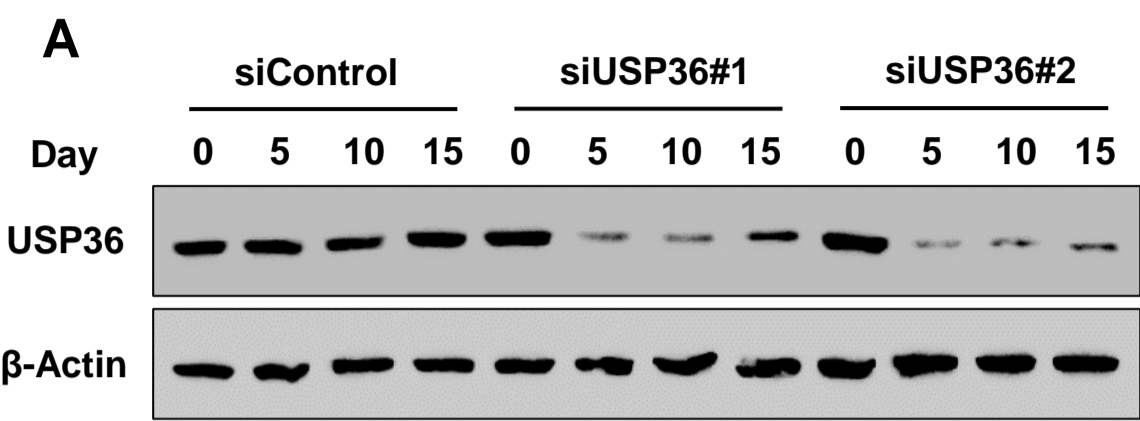

Supplement: Supplementary file 2 — Supplementary Material 2: Supplementary Figure 1: A The protein expression of USP36 in breast cancer tissues (n=125) and normal tissues (n=18) from TCGA database (https://www.genome.gov/). B: The protein expression of USP36 in ER positive breast cancer tissues (n=64) and normal tissues (n=18) from TCGA database (https://www.genome.gov/). C: Immunoblot analysis showing the expression level of USP36 in MCF-7, T47D and MCF-10A cells. β-Actin was used as the internal control. D: Real-time PCR results of USP36 mRNA expression in MCF-7, T47D and MCF-10A cells exposed as indicated. Three independent experiments were conducted to obtain the results shown in Panels C-D. All the data are presented as the means ± SDs. ***P < 0.001 for comparisons (Student’s t test). Supplementary Figure 2. A: Immunoblot analysis showing the expression level of USP36 in MCF-7 cells stably transduced with indicated shRNAs by lentivirus. Immunoblotting analyses were performed using the indicated antibodies. β-Actin was used as the internal control. B: The CCK-8 assays were used to detect the cell viability of MCF-7 cells stably transduced with indicated shRNAs by lentivirus at the indicated time points. C-D: Colony formation (left panel) of MCF-7 cells stably transduced with indicated shRNAs by lentivirus. E shows the quantitative analysis of the colony formation assay results. E-F: Transwell assays were used to detect migration ability of MCF-7 cells stably transduced with indicated shRNAs by lentivirus. G shows the quantitative analysis of the Transwell assays results. G-H: Wound healing assays were used to detect migration ability of MCF-7 cells stably transduced with indicated shRNAs by lentivirus. I shows the quantitative analysis. I-J: The percentage of apoptotic cells was determined by FACS analysis of MCF-7 cells stably transduced with indicated shRNAs by lentivirus. PI and Annexin V staining were performed on the cells. K shows the quantitative analysis. K: GREB1, PKIB, PS2 and PDZK1 mRN [file 13046_2024_3160_MOESM2_ESM.pdf]
